# Stress, Stress Management, and Dementia: A Narrative Review

**DOI:** 10.7759/cureus.94401

**Published:** 2025-10-12

**Authors:** Arunima Chaudhuri, Dharmendra K Gupta

**Affiliations:** 1 Physiology, Burdwan Medical College, Burdwan, IND; 2 Physiology, Institute of Health Professions Education, Sri Balaji Vidyapeeth, Puducherry, IND

**Keywords:** caregivers, cognitive behavioural therapy, dementia, mindfulness, prevention, stress, stress management

## Abstract

Dementia represents a growing global health crisis, with age and genetics serving as primary risk factors alongside increasingly recognized modifiable contributors. Among these, chronic stress has gained attention as a significant factor that may accelerate cognitive decline and increase dementia risk. The biological mechanisms linking stress to dementia involve multiple pathways. Persistent stress can disrupt the body's stress response system, leading to elevated stress hormones like cortisol. This chronic elevation may promote brain inflammation, cause oxidative damage to brain cells, and impair blood flow to the brain. These processes particularly affect memory-related brain regions and can contribute to the neurodegeneration characteristic of dementia. Population studies have consistently observed associations between midlife stress, depression, and psychosocial adversity with increased dementia risk later in life, sometimes decades after the initial stressful experiences. These findings suggest that the timing of stress exposure may be particularly important, with midlife representing a critical period for long-term brain health. Intervention research, while still developing, shows promise for stress-reduction approaches in preserving cognitive function. Studies of mindfulness practices, cognitive behavioral therapy, comprehensive lifestyle interventions, and caregiver support programs have demonstrated improvements in stress levels, mood, psychological resilience, and certain biological markers. Some trials have also shown modest cognitive benefits, though the evidence remains preliminary. Current limitations in stress-reduction research include relatively short study durations, small sample sizes, and questions about how well findings apply across different populations and cultural contexts. Additionally, more research is needed to determine optimal intervention timing, duration, and delivery methods. The available evidence suggests that chronic stress represents a significant and potentially modifiable risk factor for dementia. This understanding supports incorporating stress assessment and management into routine healthcare and public health approaches as a practical, cost-effective strategy for cognitive health preservation. Future priorities include developing culturally appropriate stress-reduction programs, exploring digital delivery methods for broader accessibility, and conducting longer-term studies across diverse populations to establish stress reduction as a cornerstone of dementia prevention efforts.

## Introduction and background

Dementia has emerged as one of the greatest global health challenges of the 21st century, not only due to its devastating clinical impact on patients and families but also because of its rising prevalence and economic implications. According to the World Health Organization (WHO), more than 55 million people worldwide currently live with dementia, a figure projected to nearly triple by 2050, reaching 152 million cases [1]. This demographic surge is driven primarily by population aging, particularly in low- and middle-income countries (LMICs), where health systems are often ill-prepared to manage the long-term care needs of individuals with cognitive decline [2]. The economic burden is equally staggering: annual global costs associated with dementia, including direct medical care, long-term social support, and informal caregiving, are estimated to surpass $1.3 trillion by 2030 [3].

While pharmacological research has advanced our understanding of amyloid, tau, and neurodegenerative cascades, effective disease-modifying therapies remain elusive [4]. Consequently, public health emphasis has shifted toward risk reduction and prevention strategies, targeting modifiable factors that might delay onset or reduce incidence. Among well-established modifiable risk factors are hypertension, diabetes, obesity, smoking, physical inactivity, and social isolation [5]. However, an emerging body of research highlights another, often underappreciated contributor: chronic psychological stress.

Stress is a universal human experience, shaped by occupational demands, caregiving responsibilities, socioeconomic inequalities, and health challenges. While acute stress can be adaptive, mobilizing energy and sharpening attention, chronic stress exerts deleterious effects on the brain and body. Prolonged activation of stress pathways disrupts neuroendocrine regulation, weakens immune defences, and promotes vascular injury [6,7]. Importantly, these processes overlap with core pathological features of dementia, such as hippocampal atrophy, impaired synaptic plasticity, amyloid accumulation, tau hyperphosphorylation, and cerebrovascular compromise [8,9].

The concept of allostatic load provides a framework for understanding how repeated stress exposure contributes to cumulative biological wear and tear [10]. Under chronic stress, dysregulated hypothalamic-pituitary-adrenal (HPA) axis activity leads to sustained cortisol elevations, which damage neurons and impair memory circuits. At the same time, sympathetic nervous system hyperactivity fosters hypertension and metabolic dysfunction, while systemic inflammation accelerates neurodegeneration [11]. Allostatic overload thus positions stress as not just a psychosocial phenomenon but a biological driver of cognitive decline.

One population that illustrates the cognitive consequences of chronic stress is dementia caregivers. Studies consistently show that caregivers experience elevated psychological stress, sleep disruption, and immune dysregulation, which in turn increase their risk of depression, cardiovascular disease, and cognitive impairment [12,13]. Caregiving thus acts as a “natural experiment,” demonstrating how prolonged psychosocial strain can accelerate biological aging and potentially increase dementia risk in otherwise healthy individuals. This line of research underscores the bidirectional relationship between stress and dementia: while dementia caregiving induces chronic stress, stress itself may predispose to dementia development.

Stress and its consequences are not distributed evenly across populations. Socioeconomic adversity, gender roles, cultural norms, and healthcare access shape both exposure to stressors and vulnerability to their effects. For example, women, who bear disproportionate caregiving responsibilities worldwide, face higher lifetime exposure to stress and, correspondingly, higher dementia risk [14]. Similarly, rapid social change and resource disparities in regions such as the Western Pacific have been identified as amplifiers of dementia burden, with stress functioning as a mediating factor [15]. These disparities highlight the importance of considering equity and cultural adaptation in dementia prevention frameworks.

Given the rapid accumulation of evidence, a narrative review offers the flexibility to synthesize findings across diverse research domains, including mechanistic biology, epidemiology, and clinical intervention. Recent large-scale cohort studies (e.g., Whitehall II, UK Biobank, and Swedish registries) provide robust longitudinal evidence linking chronic stress to dementia incidence [16,17]. Parallel advances in neuroimaging and biomarker research now allow more precise measurement of stress-related brain changes [18]. Moreover, randomized controlled trials (RCTs) of mindfulness, cognitive-behavioral therapy (CBT), multidomain lifestyle programs, and caregiver interventions offer preliminary but promising evidence that stress management may improve cognitive resilience and slow decline [19,20].

By integrating these mechanistic, epidemiological, and interventional findings, this review aims to evaluate the evidence base for chronic stress as a modifiable risk factor for dementia by synthesizing mechanistic, epidemiological, and interventional findings. It considers how chronic stress influences neurobiology, how it predicts dementia at the population level, and whether interventions targeting stress can mitigate cognitive decline. Beyond scientific synthesis, the review highlights clinical and public health implications, including the potential for stress assessment to become a routine part of dementia risk stratification and prevention.

## Review

Mechanistic pathways linking stress and dementia

The link between chronic stress and dementia is best explained through the biological cascades that translate psychosocial adversity into long-term neural vulnerability. Four interconnected pathways stand out: HPA axis dysregulation, neuroinflammation, oxidative stress, and vascular dysfunction.

Each contributes uniquely but also interacts with the others, ultimately creating a self-reinforcing spiral of neurodegeneration.

HPA Axis Dysregulation and Cortisol Toxicity

The HPA axis is the body’s central stress-response system, releasing cortisol to mobilize energy and maintain survival during acute challenges. However, under chronic stress, this finely tuned system becomes dysregulated. Cortisol levels remain persistently elevated, feedback inhibition is impaired, and diurnal rhythms flatten [7,21]. The hippocampus, with its dense population of glucocorticoid receptors, is particularly vulnerable to this hormonal imbalance. Sustained cortisol exposure induces dendritic retraction, reduces neurogenesis, and compromises synaptic plasticity, leading to hippocampal atrophy [11,22].

Human evidence supports this mechanistic model: Knezevic et al. (2023) showed that flattened diurnal cortisol slopes predicted dementia onset up to 10 years before diagnosis, while structural MRI studies have repeatedly linked elevated cortisol with hippocampal shrinkage and cognitive decline [18,23]. The phenomenon of “cortisol toxicity” thus illustrates how adaptive stress responses become maladaptive over time, feeding into a vicious cycle where hippocampal damage further undermines HPA regulation and increases susceptibility to cognitive impairment [10].

Neuroinflammation: Stress and Immune Dysregulation

Chronic stress also exerts a powerful influence on immune function, shifting the body from protective to pathological inflammation. Although cortisol and catecholamines initially dampen immune responses, prolonged activation paradoxically drives pro-inflammatory processes [24]. Microglia, the brain’s resident immune cells, become chronically activated, releasing cytokines such as interleukin (IL)-1β, IL-6, and tumor necrosis factor-alpha (TNF-α) that promote amyloid-β aggregation and tau hyperphosphorylation, both central features of Alzheimer’s disease [8].

Experimental models reinforce this pathway: stressed rodents show exaggerated microglial activation, increased amyloid deposition, and faster cognitive decline [25]. In humans, peripheral inflammatory markers correlate with both accelerated cognitive impairment and heightened dementia risk [26]. Moreover, chronic cortisol elevation fosters glucocorticoid resistance, reducing the body’s ability to contain inflammation [27]. This loss of regulatory control embeds neuroinflammation within the long-term trajectory of neurodegeneration.

Oxidative Stress and Mitochondrial Dysfunction

Another pathway through which stress accelerates cognitive decline is oxidative stress, arising when reactive oxygen species (ROS) overwhelm antioxidant defences. The brain is particularly vulnerable, given its high metabolic demand and lipid-rich structure. Chronic stress amplifies ROS production by disrupting mitochondrial respiration, generating free radicals through catecholamine metabolism, and activating inflammatory cascades [28]. The resulting oxidative injury damages lipids, proteins, and DNA, impairing neuronal integrity and synaptic function [9].

These molecular processes are not only destructive in themselves but also exacerbate Alzheimer’s pathology: ROS promote amyloid aggregation and tau hyperphosphorylation, creating a feed-forward cycle of degeneration [29]. Clinical studies mirror these findings, with biomarkers such as 8-OHdG (DNA oxidation) and F2-isoprostanes (lipid peroxidation) elevated in individuals under chronic stress, correlating with faster cognitive decline [30].

Vascular Dysfunction: Stress and Cerebrovascular Aging

The role of vascular health in dementia is increasingly recognized, and stress is a potent disruptor of cerebrovascular integrity. Sympathetic nervous system hyperactivity elevates blood pressure and vascular shear stress, while cortisol dysregulation fosters endothelial dysfunction and insulin resistance. Over time, these processes accelerate atherosclerosis, arterial stiffness, and microvascular rarefaction, undermining cerebral perfusion [31].

The consequences are visible in neuroimaging: white matter hyperintensities, lacunar infarcts, and microinfarcts are strongly associated with cognitive impairment and dementia risk [32,33]. Stress-related cardiovascular disease and hypertension, therefore, act as indirect yet critical mediators of dementia risk [16]. Importantly, evidence shows that antihypertensive treatment reduces dementia incidence, underscoring stress as both a direct and indirect contributor to brain aging.

Converging Mechanisms and the Neurodegenerative Spiral

Although each pathway can be described individually, their interdependence is striking. Cortisol toxicity weakens hippocampal control of the HPA axis, heightening stress reactivity. Neuroinflammation accelerates oxidative stress, which in turn fuels further inflammatory activation. Vascular injury reduces cerebral perfusion, compounding both oxidative and inflammatory damage. Together, these processes converge into a self-perpetuating “neurodegenerative spiral” that slowly erodes brain resilience. Importantly, this biological cascade often begins decades before dementia symptoms appear, highlighting the need for early identification and stress management as a preventive strategy.

Epidemiological evidence

Mechanistic studies offer strong biological plausibility for stress as a dementia risk factor, but the crucial test lies in population-level evidence. Over the last two decades, large-scale longitudinal cohorts and nationwide registries have provided increasingly consistent findings: chronic stress in midlife predicts dementia incidence decades later, even after accounting for vascular, psychiatric, and lifestyle confounders.

The Whitehall II Study

The Whitehall II cohort, which has followed more than 10,000 British civil servants for over two decades, provides some of the most influential evidence. Sommerlad et al. (2020) demonstrated that individuals reporting high psychological distress had a 40% higher risk of dementia, independent of vascular risk factors, socioeconomic status, and lifestyle behaviors [16]. Importantly, this association was not merely cross-sectional but persisted across long-term follow-up, strengthening the case for stress as a predictor rather than a consequence of cognitive decline.

Subsequent analyses from the same cohort revealed how stress contributes both directly and indirectly to dementia. Ben Hassen et al. (2022) found that early-onset multimorbidity, often linked to chronic stress, was a strong predictor of dementia incidence over 30 years [34]. This suggests that stress does not act in isolation but also fosters comorbidities such as hypertension, diabetes, and depression, which themselves accelerate cognitive decline. Together, the Whitehall II findings illustrate how stress operates as both a primary and secondary pathway in dementia risk.

Swedish Nationwide Register Studies

Sweden’s comprehensive healthcare registries offer another powerful lens into stress and dementia risk. In a cohort of more than 1.5 million adults, Wallensten et al. (2023) reported that individuals diagnosed with stress-related disorders, including post-traumatic stress disorder (PTSD) and adjustment disorder, had significantly higher dementia incidence compared with matched controls [17]. Crucially, these associations persisted even after adjusting for depression and psychiatric comorbidities, highlighting that stress itself contributes uniquely to dementia risk.

Long-term studies of women further emphasize sex-specific vulnerabilities. Guo et al. (2024), drawing on the Gothenburg Women’s Study with over 50 years of follow-up, found that midlife stress-related exhaustion was a strong predictor of late-life dementia [14]. Given that women worldwide disproportionately shoulder caregiving responsibilities and occupational stress, these findings underscore the importance of gender-sensitive approaches in dementia prevention.

Evidence From the UK Biobank

The UK Biobank, which has recruited over 500,000 participants, further reinforces the association between stress and cognitive aging. Hendriks et al. (2024) reported that psychological distress and sleep disruption, common consequences of chronic stress, were linked to a higher risk of cognitive impairment and dementia [35]. Similarly, Jin et al. (2025) showed that adult education and lifelong learning, which reflect resilience and cognitive reserve, were associated with larger brain volumes and reduced dementia incidence [36]. These findings suggest that while stress increases vulnerability, protective factors such as resilience behaviors and education can buffer its effects.

The Role of Vascular Stressors

Stress also contributes indirectly to dementia through its impact on vascular health. Hypertension, one of the most common stress-related conditions, has repeatedly been shown to accelerate cognitive decline. A recent meta-analysis confirmed that hypertension significantly increases dementia risk, while antihypertensive treatment reduces incidence [37]. Since chronic stress exacerbates hypertension through sympathetic overactivity and cortisol dysregulation, this establishes stress as a dual risk factor: directly through brain biology and indirectly via vascular disease [33].

Global Evidence Beyond Europe

Although much of the strongest data originates from European cohorts, studies from other regions highlight the universality of this association. In the United States, the Health and Retirement Study found that older adults with high perceived stress and depressive symptoms were significantly more likely to develop dementia [38]. The study by Sindi et al. followed 1,511 Finnish adults for 25 years (from age 50 to 78 years) to examine how midlife work-related stress affects late-life cognitive function in people without dementia. Higher midlife work stress was associated with poorer global cognition and slower processing speed in later life, even after accounting for other factors. Work stress did not significantly affect memory, executive function, verbal fluency, or manual dexterity, suggesting that global cognition and processing speed are most vulnerable to midlife workplace stress effects [39]. Research in Latin America points to the combined impact of psychosocial stress and limited educational attainment on dementia risk, while studies in the Western Pacific emphasize how social change and economic disparities amplify dementia burden, with stress as a mediating factor [15,40].

Meta-Analyses and Systematic Reviews

Several meta-analyses and systematic reviews have strengthened the evidence linking psychological factors such as depression and stress with dementia risk. Fernández et al. (2024) conducted a comprehensive meta-analysis of 26 longitudinal studies, including 1,760,262 participants, to investigate depression as a risk factor for dementia. Their pooled analysis yielded a relative risk (RR) of 1.82 (95% CI = 1.62-2.06), indicating that individuals with depression have almost twice the risk of developing dementia compared to those without depression [41]. The findings were consistent across different diagnostic methods for both depression and dementia, suggesting a robust and clinically significant association that persists irrespective of the diagnostic approach used. This meta-analysis highlights the potential benefit of early identification and effective management of depressive symptoms as part of dementia prevention strategies.

Complementing these results, Franks et al. (2021) conducted a systematic review and meta-analysis assessing the role of psychological stress in cognitive decline. Across multiple prospective cohort studies, they reported that higher levels of stress were significantly associated with increased risk of both dementia and mild cognitive impairment (MCI). The pooled results showed a hazard ratio (HR) of approximately 1.33 (95% CI ≈ 1.18-1.49) for dementia, emphasizing that chronic stress contributes meaningfully to neurodegenerative risk, likely via HPA axis dysregulation, glucocorticoid neurotoxicity, and inflammatory pathways [42]. Together, these two studies consolidate the growing body of evidence that both depression and stress are modifiable psychosocial risk factors, offering a window for early interventions that may mitigate long-term dementia risk.

Limitations of Epidemiological Evidence

Despite the strength of these findings, limitations remain. Reverse causality is difficult to fully rule out, as early cognitive decline may itself increase perceived stress. Stress measurement is inconsistent across studies, ranging from self-report scales to clinical diagnoses and biomarkers, complicating comparability. Residual confounding from lifestyle, genetics (e.g., APOE ε4), and socioeconomic status cannot be excluded, and much of the data comes from high-income countries, limiting global generalizability. These caveats underscore the need for large, diverse, biomarker-integrated prospective studies to establish causality with greater certainty.

Taken together, epidemiological evidence strongly supports the role of stress as an independent and modifiable risk factor for dementia. Longitudinal data across Europe, North America, Asia, and Latin America consistently show that midlife stress predicts dementia risk decades later, while protective factors such as resilience and cognitive reserve can buffer these effects. Stress, therefore, emerges not as a peripheral correlate but as a central determinant of cognitive aging, justifying its integration into dementia prevention frameworks worldwide.

Interventional evidence

While epidemiological studies highlight the risks posed by chronic stress, the critical question is whether stress reduction can alter the trajectory of cognitive decline. Interventions serve as real-world tests of causality: if managing stress improves resilience, biomarkers, or cognition, it strengthens the case for stress as a modifiable risk factor. Recent years have seen increasing attention to mindfulness, CBT, multidomain lifestyle approaches, and caregiver-targeted programs. Although evidence remains preliminary, RCTs suggest that stress management is feasible, beneficial, and adaptable across populations.

Mindfulness-Based Interventions

Mindfulness-based stress reduction (MBSR) and related meditation practices have been studied for their ability to modulate both psychological well-being and biological stress pathways. Moye (2025), in a review of 20 RCTs involving older adults, including those with MCI, reported improvements in cortisol regulation, heart rate variability, emotional resilience, and modest cognitive gains. Importantly, neuroimaging studies demonstrated increased cortical thickness in regions tied to executive control, suggesting that mindfulness may enhance brain reserve. However, adherence was challenging, particularly among participants with early cognitive symptoms [43].

Earlier pilot work by Brown et al. (2016) provided mechanistic evidence by showing that mindfulness meditation improved memory, reduced anxiety, and altered brain connectivity patterns in older adults with anxiety and cognitive complaints. While small in scale, this study reinforced the idea that stress reduction directly influences brain function [44]. Taken together, mindfulness interventions appear safe, low-cost, and scalable, though larger and longer trials are needed to confirm their preventive effects on dementia.

Cognitive Behavioral Therapy (CBT)

CBT, with its structured focus on reframing maladaptive thoughts and improving coping skills, has also been tested in older populations. Jeste (2025) found that CBT reduced perceived stress, improved executive functioning, and lowered cortisol levels in late-life stress disorders [45]. These outcomes demonstrate that CBT can address both psychological symptoms and biological stress markers.

Earlier evidence by Gould et al. (2012) in geriatric patients with depression showed improvements in mood and resilience, although cognitive benefits were modest and limited by sample size [46]. These findings highlight CBT’s potential as a dual-acting intervention, addressing mood and stress-related biology, but also its challenges: the need for trained therapists and cultural adaptation. Scalable models such as group-based or digital CBT platforms may help overcome these barriers.

Multidomain Lifestyle Programs

Because dementia is multifactorial, interventions that combine diet, exercise, cognitive training, and stress management are particularly promising. The AgeWell.de trial (Zülke et al., 2025) demonstrated that a two-year multidomain program in older adults at increased genetic risk improved composite dementia risk scores, depressive symptoms, and resilience behaviors [20]. Stress reduction strategies were embedded alongside dietary and physical activity changes, making it difficult to isolate their independent contribution, but the program nonetheless illustrated the power of integrated prevention.

The landmark FINGER trial by Ngandu et al. (2015), which recruited 1,260 at-risk older adults in Finland, further showed that multidomain interventions significantly improved cognition and executive function after two years. Although stress management was not an explicit focus, the program’s lifestyle and social engagement components indirectly reduced psychosocial stress, providing compelling evidence that multifaceted approaches can delay cognitive decline [19].

Caregiver Interventions

Caregivers of dementia patients are uniquely vulnerable to chronic stress, making them both a target for support and a natural experiment in stress-related cognitive risk. Interventions for caregivers thus serve dual purposes: protecting caregivers’ health and indirectly improving patient care.

Silva et al. (2025) tested Engage Coaching, a culturally adapted, CBT-based program for Latino dementia caregivers. Results showed improvements in resilience, reduced loneliness, and modest cognitive benefits, underscoring the importance of cultural tailoring. Evidence for stress-management interventions in caregivers is strengthened by RCTs of mindfulness-based approaches. Whitebird et al. (2013) evaluated an eight-week MBSR program in 78 family caregivers of persons with chronic conditions. Compared with a wait-list control group, participants in the MBSR group demonstrated significant improvements in perceived stress, depression, and anxiety, along with better mental health-related quality of life at follow-up. Although cognitive outcomes were not measured as primary endpoints, these psychological improvements are clinically meaningful and may indirectly support cognitive health and reduce long-term dementia risk by lowering chronic stress exposure and HPA axis dysregulation [47,48]. Such findings, combined with culturally tailored CBT programs like Engage Coaching, reinforce the dual value of supporting caregivers’ mental health and potentially reducing their future dementia risk.

Synthesis of RCT evidence

Across the eight RCTs reviewed (Table 1), several patterns emerge. Mindfulness and CBT consistently reduce perceived stress and show modest cognitive benefits. Multidomain lifestyle trials provide the strongest cognitive outcomes, though stress is often embedded rather than isolated as a target. Caregiver interventions highlight the dual value of protecting a high-risk group while advancing health equity.

**Table 1:** Key findings and comparison of the strengths, limitations, and suggestions for the eight RCTs included in this narrative synthesis. RCT: randomized controlled trial; LMICs: low- and middle-income countries; PSS: Perceived Stress Scale; POMS: Profile of Mood States; SF-12: 12-item Short Form Survey; HRV: heart rate variability.

Study (year)	Population	Sample size/age range	Intervention	Duration	Primary outcomes	Secondary outcomes	Strengths	Limitations	Suggestions
Ngandu et al. (2015) (FINGER) [19]	At-risk older adults (Finland)	n = 1,260; age = 60–77 years	Multidomain intervention: diet, exercise, cognitive training, vascular risk management	2 years	Cognitive function (comprehensive neuropsychological battery)	Depression, vascular risk factors, and daily functioning	Landmark, first, large multidomain dementia prevention RCT; strong methodology and long follow-up	Resource-intensive, stress not directly measured	Incorporate stress biomarkers in future multidomain trials; replicate in diverse, low-resource populations
Zülke et al. (2025) (AgeWell.de) [20]	Older adults at increased genetic risk of dementia	n = 1,050; age = 60–77 years	Multidomain lifestyle program (diet, activity, cognitive training, stress management)	24 months	Composite dementia risk score	Dietary habits, depressive symptoms, and adherence	Large, well-powered, European multicenter trial with long follow-up	Stress is not isolated as a primary endpoint, culturally specific to Germany	Replicate in LMICs; subgroup analyses to isolate the effect of stress management
Moye (2025) [43]	Community-dwelling older adults, including some with mild cognitive impairment (MCI)	n ≈ 180; mean age ≈ 70 years	Group-based mindfulness-based stress reduction (MBSR)	8 weeks + 3-month follow-up	Perceived stress, diurnal cortisol slope, and executive function	Hippocampal volume (MRI), resilience scores	Innovative integration of psychosocial outcomes, stress biomarkers, and neuroimaging	Short duration, moderate attrition, limited to motivated participants	Extend trial to ≥1 year, assess dementia incidence as outcome, test digital MBSR delivery for scalability
Brown et al. (2016) [44]	Family caregivers of persons with dementia (high stress group)	n = 38; mean age ≈ 65 years	Mindfulness-based stress reduction (MBSR)	8 weeks	Perceived stress (PSS), mood disturbance (POMS)	Salivary cortisol (diurnal slope), sleep quality	Included a biological stress marker (cortisol) to explore the mechanistic pathway	Small pilot study, underpowered for cognition, limited follow-up	Replicate with larger, multicenter cohorts; integrate cognitive endpoints and long-term biomarker follow-up
Jeste (2025) [45]	Older adults with late-life stress disorders	n ≈ 150; age = 65–80 years	Individual cognitive behavioral therapy (CBT)	12 weeks + 6-month follow-up	Perceived stress, executive function	Salivary cortisol, depression severity, quality of life	Demonstrated feasibility and efficacy of CBT for stress reduction in older adults	Requires trained therapists, less scalable, cultural adaptation needed	Develop group-based or digital CBT models; include diverse populations and biomarkers
Gould et al. (2012) [46]	Geriatric patients with depressive symptoms	n = 80; age ≥ 60 years	Weekly CBT sessions	12 weeks	Depression severity, perceived stress	Cognitive performance, sleep quality	Provided validated psychological outcome data in older adults	Cognitive outcomes underpowered, biomarkers missing, small sample size	Include biological stress markers, larger sample, longer follow-up
Silva et al. (2025) (Engage Coaching) [47]	Latino dementia caregivers (high stress group)	n = 90; mean age ≈ 62 years	Engage Coaching (CBT-based, culturally tailored)	6 months	Caregiver burden, perceived stress	Sleep quality, depressive symptoms	Strong cultural tailoring; feasibility and acceptability demonstrated	Modest sample, cognitive outcomes secondary, biomarkers lacking	Include biological endpoints and replication in other ethnic groups
Whitebird et al. (2013) [48]	Family caregivers of persons with chronic conditions	n = 78; mean age ≈ 60 years	Mindfulness-based stress reduction (MBSR)	8 weeks	Perceived stress, depression, anxiety	Mental health-related quality of life (SF-12)	Well-designed RCT; significant improvements in psychological outcomes; feasible group format	Short follow-up, no cognitive endpoints, no biomarkers	Replicate with biomarker integration (cortisol, HRV) and cognitive follow-up; test digital delivery for scalability

Limitations remain clear: most trials are short-term studies (≤ three years), with modest sample sizes and limited biomarker integration. Stress management components are often secondary to broader lifestyle changes, and generalizability is constrained by overrepresentation of Western, urban cohorts. To establish stress reduction as a pillar of dementia prevention, future RCTs will need longer follow-up, biomarker endpoints, culturally diverse samples, and scalable delivery platforms.

Clinical and Public Health Implications

The convergence of mechanistic, epidemiological, and interventional evidence makes a strong case for chronic stress as a modifiable risk factor for dementia. Translating this knowledge into practice requires a multipronged approach that spans clinical care, caregiver support, health equity, and policy frameworks.

Stress Screening in Dementia Risk Assessment

Routine stress assessment should be incorporated into dementia risk profiling alongside established measures such as blood pressure, glucose, lipid panels, and cognitive testing. Validated tools like the Perceived Stress Scale (PSS) or the Caregiver Strain Index are quick, inexpensive, and clinically feasible [49]. Complementing self-reports with biomarkers, including diurnal cortisol slopes, heart rate variability, and inflammatory cytokines, may enhance risk stratification by capturing biological stress load [50].

Importantly, screening is most valuable in midlife, when stress exposures exert the strongest long-term influence on dementia risk. Embedding stress evaluation into primary care visits and memory clinics would represent a practical and cost-effective step toward prevention.

Addressing Caregivers as a High-Risk Group

Dementia caregivers exemplify the intersection between psychosocial stress and biological vulnerability. Evidence shows that caregiving stress contributes not only to depression and immune dysfunction but also to accelerated biological aging, including telomere shortening and higher dementia risk [13]. Interventions such as CBT-based coaching, mindfulness, and structured support groups have demonstrated effectiveness in reducing caregiver stress and improving resilience [47].

Supporting caregivers thus has dual benefits: improving their own well-being while also enhancing the quality of care provided to patients. Policymakers should recognize caregiver health as a critical public health priority and allocate resources to sustainable caregiver support programs as part of national dementia strategies.

Equity and Cultural Adaptation

The burden of stress and dementia is shaped profoundly by social and cultural contexts. Women, ethnic minorities, and individuals in low-resource settings often face disproportionately high stress exposures due to caregiving responsibilities, socioeconomic adversity, and limited healthcare access [15]. Ignoring these disparities risks widening inequities in dementia outcomes.

Culturally adapted programs, such as Silva et al.’s Engage Coaching for Latino caregivers, demonstrate that tailoring interventions enhances both feasibility and effectiveness [47]. Public health strategies must therefore adopt an equity lens, ensuring interventions are not only clinically sound but also accessible and culturally resonant across diverse populations.

Integration Into National Dementia Prevention Frameworks

National dementia prevention strategies increasingly highlight the importance of modifiable risk factors [5]. Stress management should be explicitly incorporated alongside priorities such as hypertension control, physical activity, and social engagement. Given its low cost and wide applicability, stress reduction offers a scalable preventive measure with potential for broad population impact.

Digital technologies further enhance scalability. Wearables and mobile applications can track stress-related biomarkers such as heart rate variability, while delivering personalized mindfulness or CBT-based modules through telehealth platforms. These tools are particularly relevant for LMICs, where healthcare infrastructure is limited and digital solutions may provide the most practical means of intervention.

Clinical and public health efforts to manage stress represent a rare opportunity in dementia prevention: they are cost-effective, broadly applicable, and synergistic with existing lifestyle and vascular interventions. By embedding stress screening into clinical practice, supporting caregivers, prioritizing cultural adaptation, and leveraging digital technologies, healthcare systems can move toward proactive strategies that preserve cognitive health and reduce the global dementia burden.

Future directions

Although the evidence base strongly supports stress as a modifiable dementia risk factor, several gaps remain. Addressing these challenges will be essential to establishing stress reduction as a cornerstone of dementia prevention.

Large-Scale, Long-Term RCTs

Most existing RCTs are limited by modest sample sizes, short follow-up (≤ three years), and reliance on Western, urban populations. Dementia prevention, however, requires decades of sustained observation. Future trials should recruit diverse cohorts across cultural and socioeconomic settings, follow participants for 10-20 years, and include biological, psychological, and cognitive outcomes [19,20]. Importantly, stress should be tested as a primary intervention target, not just a secondary component embedded within lifestyle programs. Such studies would parallel landmark lifestyle trials like FINGER but with an explicit focus on stress management.

Biomarker Development

Validated biomarkers are critical for translating stress research into clinical practice. Cortisol dysregulation, inflammatory cytokines (e.g., IL-6 and TNF-α), oxidative stress markers such as F2-isoprostanes and 8-OHdG, and neuroimaging measures of hippocampal atrophy or white matter hyperintensities represent promising candidates [18,30]. Establishing reliable biomarker panels would enable early identification of high-risk individuals, personalized risk stratification, and precise monitoring of intervention efficacy.

Understanding Resilience Mechanisms

Not all individuals exposed to chronic stress develop dementia, suggesting the presence of resilience factors. Social support, education, physical activity, and cognitive reserve may buffer against stress-related neurobiological damage [36]. Unpacking these protective mechanisms could inform targeted prevention strategies that build resilience rather than simply reduce stress exposure. Future studies should investigate how genetic and environmental modifiers interact to determine individual vulnerability or resistance.

Digital Health and Precision Prevention

Wearables, mobile applications, and artificial intelligence (AI) platforms offer transformative potential for stress management. Continuous monitoring of physiological stress markers, such as heart rate variability, skin conductance, and sleep patterns, could allow real-time detection of stress surges. AI-driven tools could then deliver personalized interventions (e.g., mindfulness exercises, guided breathing, or digital CBT) precisely when individuals need them. By embedding stress management into daily life, digital health solutions could shift dementia prevention from reactive care to proactive precision prevention [28].

Summary of evidence classification by strength and validity

The evidence linking stress to dementia can be classified into three hierarchical categories based on methodological rigor and validity.

Mechanistic Evidence (Moderate Strength)

The biological pathways connecting chronic stress to dementia, including HPA axis dysregulation, neuroinflammation, oxidative stress, and vascular dysfunction, demonstrate strong biological plausibility supported by experimental models and biomarker studies. However, most mechanistic data derive from animal models and cross-sectional human studies, limiting causal inference. Cortisol toxicity shows particularly robust evidence, with flattened diurnal cortisol slopes predicting dementia up to 10 years before diagnosis.

Epidemiological Evidence (Strong-to-Moderate Strength)

Large-scale longitudinal cohorts, including Whitehall II, Swedish nationwide registries, and UK Biobank, provide consistent evidence that midlife stress predicts dementia decades later, independent of confounders. Meta-analyses strengthen this association, with depression showing a relative risk of 1.82 for dementia and chronic stress yielding a hazard ratio of approximately 1.33. However, limitations include potential reverse causality, inconsistent stress measurement across studies, possible residual confounding from genetics and socioeconomic factors, and overrepresentation of high-income countries.

Interventional Evidence (Preliminary-to-Moderate Strength)

RCTs of mindfulness, CBT, and multidomain lifestyle interventions demonstrate feasibility and show improvements in stress biomarkers, mood, and modest cognitive benefits. However, most trials suffer from short durations (≤ three years), modest sample sizes, and limited biomarker integration. Landmark studies provide the strongest interventional evidence, though stress management was often embedded rather than isolated as a primary target.

The evidence progresses from strong biological plausibility through robust observational associations to preliminary but promising interventional data, collectively supporting stress as a modifiable dementia risk factor requiring further confirmatory research.

## Conclusions

Stress represents one of the most pervasive yet underrecognized threats to brain health across the human lifespan. The biological mechanisms are clear: chronic stress triggers a cascade of harmful processes, including brain tissue loss, widespread inflammation, cellular damage, and impaired blood flow to critical brain regions. Population-level evidence consistently demonstrates that individuals experiencing significant stress during middle age face substantially higher rates of cognitive decline and dementia in later years. Importantly, stress is not an inevitable burden we must endure. Multiple approaches have proven effective at reducing stress levels, from mindfulness practices and CBT to lifestyle modifications and enhanced social support systems. These interventions consistently show measurable benefits for both psychological well-being and cognitive function. The time has come to fundamentally reframe how we view stress in healthcare. Rather than treating it as an unavoidable aspect of modern life or a purely psychological concern, stress should be recognized as a modifiable biological risk factor for dementia. This shift in perspective opens new possibilities for prevention strategies that are both practical and cost-effective. Incorporating stress assessment and management into routine healthcare and broader public health initiatives represents a promising pathway forward. Such approaches could reach many people relatively cheaply, potentially delaying or preventing countless cases of cognitive decline. Every step taken to reduce stress in our communities is simultaneously an investment in protecting cognitive health and enhancing quality of life for aging populations worldwide.
